# The Influence of Grape Clone and Yeast Strain on Varietal Thiol Concentrations and Sensory Properties of Graševina Wines

**DOI:** 10.3390/foods12050985

**Published:** 2023-02-26

**Authors:** Marina Tomašević, Katarina Lukić, Natka Ćurko, Ana-Marija Jagatić Korenika, Darko Preiner, Valentina Tuščić, Ana Jeromel, Karin Kovačević Ganić

**Affiliations:** 1 Faculty of Food Technology and Biotechnology, University of Zagreb, Pierottijeva 6, 10000 Zagreb, Croatia; 2 Faculty of Agriculture, University of Zagreb, Svetošimunska 25, 10000 Zagreb, Croatia; 3 Centre of Excellence for Biodiversity and Molecular Plant Breeding, Svetošimunska 25, 10000 Zagreb, Croatia

**Keywords:** clonal selection, *Saccharomyces* yeasts, *Metschnikowia pulcherrima*, white wine, varietal aroma

## Abstract

Varietal thiol concentration in wine is influenced by numerous factors, of which grape variety and winemaking practices are often highlighted as the most important. Therefore, the aim of this work was to study the effects of grape clone and yeast strain (*Saccharomyces* and non-*Saccharomyces*) on the varietal thiols concentrations and sensory characteristics of Graševina (*Vitis vinifera* L.) white wines. Two grape clones were evaluated (OB-412 and OB-445) along with three different commercial yeast strains (*Saccharomyces cerevisiae* Lalvin Sensy and Sauvy, and *Metschnikowia pulcherrima* Flavia). The results showed that the concentration of varietal thiols in Graševina wines amounted up to a total of 226 ng/L. The clone OB-412 was characterized by its significantly higher concentrations, especially of 3-sulfanylhexanol (3SH) and 3-sulfanylhexyl acetate (3SHA). Moreover, alcoholic fermentation with pure *S. cerevisiae* Sauvy yeasts generally resulted in higher thiol concentrations, while sequential fermentation involving *M. pulcherrima* positively affected only the 4-methyl-4-sulfanyl-pentan-2-one (4MSP) concentration. Finally, sensory analysis showed that fermentation with pure *S. cerevisiae* Sauvy yeast also produced more desirable wines. The results suggest that clonal, and especially yeast strain, selections are important modulators of the aroma and sensory properties of wine.

## 1. Introduction

The chemical composition and sensory characteristics of wines are strongly influenced by the volatile components released during the initial stages of winemaking and especially during alcoholic fermentation [1,2,3]. In terms of sensory properties, varietal thiols are one of the most desirable groups of aroma compounds in wines. Herein, three thiols are important: 4-methyl-4-sulfanylpentan-2-one (4MSP), 3-sulfanylhexanol (3SH) and 3-sulfanylhexyl acetate (3SHA). These compounds contribute to wine aroma by adding grapefruit, passion fruit, citrus zest and boxwood nuances [4,5]. In wines, they are present in extremely low concentrations (ng/L) with equally low sensory perception thresholds of 0.8, 60 and 4 ng/L for 4MSP, 3SH and 3SHA, respectively [4].

Although these compounds are variety dependent, there are several ways that winemakers can influence their final concentration in the wine, as well as the overall aroma composition of the wine. For example, it has been demonstrated that different clones of the same grape variety result in wines with different thiol concentrations. Most of these investigations are related to Sauvignon blanc, which is the main representative of the thiol-rich grape variety. Herein, Capone et al. [6] showed that both the concentration of varietal thiols and the thiol precursors significantly differed between five Sauvignon blanc clones. In addition, Šuklje et al. [7] concluded that the concentrations of 3SH and 3SHA were also different between investigated Sauvignon blanc clones. Our recent study confirmed that clone selection is a potential modulator of varietal thiol concentration in Pošip wine [8]. Moreover, the (S):(R) ratio of chiral 3SH and 3SHA was also different between Sauvignon blanc clones [9].

Aside from clonal selection, the most common way to easily modulate wine aroma is through yeast selection. In general, the role of yeast in wine aroma formation is undisputed, as it is associated with different types of flavor-active compounds formed either de novo or because of modifications to precursor molecules from grapes [10]. Varietal thiols belong to the latter group, as they are predominantly present in conjugated, non-volatile form and are mainly bound to cysteine and glutathione [11,12]. The volatile, odoriferous components are released by enzymatic β-lyase activity produced mainly by certain wine yeasts [13]. Belda et al. [14] showed that this activity was strain dependent and several non-*Saccharomyces* species were especially emphasized. One of these was *Metschnikowia pulcherrima*, a strain that is often highlighted for its ability to reduce the alcohol content of wines [15,16], as well as for its ability to produce a natural antimicrobial agent (pulcherrimin) that has been shown to have effective inhibitory activity against spoilage yeasts and fungi [17]. In terms of wine aroma, it has been demonstrated that fermentation involving *M. pulcherrima* resulted in a higher concentration of the thiol 4MSP [15], as well as in higher fruity and floral aromas and a better overall impression of the wines produced [18,19].

Graševina (*Vitis vinifera* L.) is the most represented and the most economically important grape variety in Croatia, with almost a quarter (24%) of the total vineyards planting this variety [20]. Generally, this white grape variety is grown in Central and Eastern Europe, and its aromatic expression is considered to be strongly dependent on the planting material, geographical location, yield and grape ripeness as well as the applied winemaking procedures [21]. Intravarietal variability was detected within a population of Graševina, and the clone selection showed substantial differences between clone candidates, primarily in terms of yield, number of clusters per vine and the basic quality indicators in produced must [20].

The aim of this study was to investigate the effects of the grape clone and the yeast strain on the concentration of varietal thiols in Graševina wine, as well as on the sensory characteristics of the wines produced. For this purpose, two clones and three yeast variants (*Saccharomyces* and non-*Saccharomyces*) were studied.

## 2. Materials and Methods

### 2.1. Materials

#### 2.1.1. Chemicals

The ethanol was HPLC grade and was purchased from J.T. Baker (Deventer, The Netherlands), while the ethyl acetate, hydrochloric acid (37%) and sodium sulfate anhydrous were purchased from Carlo Erba (Val-de-Reuil, Spain). The sodium acetate trihydrate was purchased from Gram-mol (Zagreb, Croatia) and the cysteine hydrochloride hydrate, dichloromethane, *p*-hydroxymercurybenzoate (*p*-HMB), 5,5-dithio-bis (2-nitrobenzoic acid) (DTNB), Dowex exchange resin and butylated hydroxyanisole (BHA) were purchased from Sigma Aldrich (St. Louis, MO, USA). In addition, the 4-methyl-4-sulfanylpentan-2-one and 3-sulfanylhexyl acetate were purchased from Alfa Aesar (Ward Hill, MA, USA), while the 3-sulfanylhexanol and 4-methoxy-2-methyl-2-sulfanylbutane were purchased from Endeavour Chemicals (Northamptonshire, UK).

#### 2.1.2. Wines

Grapes from two registered Graševina clones that were selected from the Kutjevo wine region were used for vinification: clone OB-412 and clone OB-445. Both were grown at the experimental vineyard Jazbina, Zagreb, in the Croatian Uplands region, during the 2020 vintage. The vineyard was planted in 2015, and vines were grafted onto the Kober 5BB rootstock. Plants were grown on a vertical trellis with 2.1 m between the rows and 0.8 m between plants, and they were pruned according to the single Guyot training system. The morphological characteristics of the clone OB-445 are, generally, standard for this grape variety: average yield, average sugar and total acidity content, medium size and lower density bunch. The OB-412 clone is characterized by a slightly lower yield, high sugar content, very high total acidity (combined with high sugar content), lower weight bunch and smaller berries. This clone is particularly suitable for cultivation in the C1 zone, due to its higher acidity and sugar content [20].

An amount of 400 kg of healthy grapes from each clone was handpicked and transferred to the experimental cellar at the Department of Viticulture and Enology, Faculty of Agriculture, in Zagreb. Grapes from each clone were destemmed, crushed, and pressed, and the obtained musts were clarified for 24 h (sulfurous acid was added at a concentration of 50 mg/L total SO_2_). Each clarified must was decanted and separated into nine inox tanks (three repetitions for each variant). The control variant was inoculated with *Saccharomyces cerevisiae* yeast strain Lalvin Sensy™ (marked with the letter C) and the second was inoculated with *S. cereviae* Sauvy™ (marked with the letter S), while the third variant represented sequential fermentation with *M. pulcherrima* Flavia™ and *S. cerevisiae* Sauvy™ after 24 h (marked with the letter F) (all yeasts were purchased from Lallemand, Montreal, Canada). The yeasts were inoculated with the fermentation nutrient Stimula Sauvignon blanc™ (Lallemand, Montreal, QC, Canada), while Fermaid E™ (Lallemand, Montreal, QC, Canada) was added to the fermenting must after one-third of the alcoholic fermentation to ensure the completion of the process. After completion of alcoholic fermentation, wines were racked, the free sulfur dioxide content was corrected to 35 mg/L and the samples were frozen at −20 °C until laboratory analyses.

### 2.2. Methods

#### 2.2.1. Physicochemical Parameters of Produced Wines

The physicochemical parameters (alcohol content, reducing sugars, total extract, pH, titratable acidity (TA), volatile acidity (VA) and total and free SO_2_) of produced wines were analyzed according to the conventional wine analyses [22].

#### 2.2.2. Varietal Thiols Extraction and Analysis

The varietal thiols were extracted according to a slightly modified version of the procedure developed by Tominaga et al. [4], which is described in detail by Tomašević et al. [23]. Amounts of 0.5 mL of BHA, 5 mL of 1mM *p*-HMB and 50 μL of 4-methoxy-2-methyl-2-sulfanylbutane (internal standard) were added to 50 mL of wine. The sample was stirred, and pH was corrected to 7.0 and percolated to the exchange column (Dowex, 1 × 2, Cl^−^ form). The column was then washed with 0.1 M sodium acetate buffer at pH = 6.0, after which the thiols were released by percolating a solution of cysteine hydrochloride monohydrate (pH = 6.0) through the column. An amount of 0.5 mL of ethyl acetate was added to the eluted sample and the thiols were then extracted using two successive additions of 15 and 10 mL of dichloromethane. The recovered organic phases were dried with anhydrous sodium sulfate and then concentrated, first under vacuum and then under nitrogen flow to approximately 30 μL. The concentrated sample was injected into the GC/MS system.

Thiols were separated by a BP20 capillary column (50 m × 220 mm, 0.25 mm, SGE Analytical Science, Melbourne, Australia) using a Gas Chromatograph 6890 coupled with a 5973 Inert mass selective detector (Agilent Technologies, Santa Clara, CA, USA). The detector interface temperature was set at 250 °C and the ion source, operating in EI mode at 70 eV, was maintained at 280 °C. The injection port was heated to 250 °C, and the following temperature program was used: 35 °C, 10 min → 230 °C, 3 °C/min. Acquisition was performed in the Selective Ion Monitoring mode (SIM) with selected ions (m/z): 134 and 75 for the internal standard; 132, 75, 99 for 4MSP; 134, 100, 101 for 3SH; and 116 and 101 for 3SHA [23]. Enhanced Chemstation software (Agilent Technologies, Santa Clara, CA, USA) was used for the identification and quantification of compounds.

#### 2.2.3. Sensory Analysis

The wines were subjected to sensory analysis by seven trained judges using quantitative descriptive analysis (QDA) and a ranking test. The judges were members of the Croatian Enology Society, and all were highly experienced in wine sensory tasting. They were initially screened based on (i) sensitivity to the recognition of the basic tastes in standard solutions containing concentrations slightly higher than its thresholds and, further, on (ii) discriminant capability to determine differences in the flavor of white wine samples in the differences from control test [24]. After the screening, a training session took place, wherein a total of 17 olfactory descriptors were generated and each term was consensually defined and described [25]. In addition to defining each attribute, the training sessions also aimed to achieve an intensity rating in a reliable way. For that, olfactory and gustatory standards were adopted. The training was performed during eight training sessions (2 × 2 h sessions per week, held over four consecutive weeks). Finally, the judges developed a sensory descriptive ballot for the wines, associating each descriptor with a scale from 0 to 5 (0-absence of perception, 1-low, 2-slight, 3-moderate, 4-intensive, 5-very intensive). Wines were evaluated in duplicate according to a completely randomized design to balance presentation order and carryover effects [26]. Samples (35–40 mL) were served in clear wine-tasting glasses [27] and labeled with a three-digit code. The temperature of the wines was maintained at 15 ± 1 °C.

In the ranking test, judges were asked to rank wines in descending order of preference: value 1 was assigned to the most preferred wine, value 2 to the next most preferred wine, and value 3 to the least preferred wine [28].

#### 2.2.4. Statistical Analysis

Statistical data analysis was performed using Statistica V.12 software (Statsoft Inc., Tulsa, OK, USA). Varietal thiol data were subjected to a factorial analysis of variance (ANOVA) to determine whether there were significant differences between the clones and yeasts and to evaluate the significance of clone*yeast interactions. Tukey’s HSD test was used as a comparison test when samples differed significantly after conducting the ANOVA (*p* < 0.05).

Data from the preference ranking test were subjected to the Friedman analysis, in which the Friedman statistic (*F*) was calculated [28] and compared with a tabulated critical value that indicated the minimum value required to conclude a significant difference between two or more of the three wines tasted. A significance level of 5% was used (*p* = 0.05).

## 3. Results and Discussion

### 3.1. Physicochemical Parameters

Table 1 shows the values of the main physicochemical parameters of the produced Graševina wines. The alcohol content (vol%) varies greatly between the wines produced, with the highest value found in the wines OB-445 C and OB-445 S, followed by OB-412 S, OB-412 C, and, finally, OB-412 F and OB-445 F, which were those with the lowest alcohol content. These results indicate lower alcohol production by sequential fermentation of *M. pulcherrima* and *S. cerevisiae* (treatment F), which is consistent with previous studies wherein authors confirmed that some non-*Saccharomyces* species produce lower ethanol yields [15,29,30]. In the case of *M. pulcherrima*, Ruiz et al. [15] reported 0.6% (*v*/*v*) lower ethanol concentrations than in wines fermented by *S. cerevisiae*. In addition, Sadoudi et al. [29] reported a difference of 0.44% (*v*/*v*) between these yeast variations. In the current study, the differences were 0.3 and 0.4% (*v*/*v*) for the clones OB-412 and OB-445, respectively, and were similar to those reported in the previously mentioned study, 

In addition to the lowest alcohol contents, the wines fermented by sequential fermentation of *M. pulcherrima* and *S. cerevisiae* also had the lowest total extract and titratable acidity (TA) contents, as well as the highest volatile acidity (VA) and pH values. Previous studies have also reported that *M. pulcherrima* can reduce the total acidity of the final wines [31,32], although this effect can be considered positive or negative depending on the initial acidity of the grape juice [31]. In the case of this study, it is positive rather than negative due to the high TA, which could result in a softer mouthfeel of the final wine. Regarding VA, the results of different studies are inconsistent. For example, Comitini et al. [31] found lower VA content in wines fermented with mixed cultures of *M. pulcherrima* and *S. cerevisiae* than in wines fermented with *S. cerevisiae* alone. Sadoudi et al. [29] also demonstrated higher amounts of acetic acid in wines produced with pure *S. cerevisiae* compared to sequential fermentation with the previously mentioned yeasts, and they emphasized that the presence of *M. pulcherrima* together with *S. cerevisiae* in a culture resulted in a decrease in acetic acid production from the beginning of fermentation. This group of authors also reported similar results and trends in their previous study [33]. On the other hand, similar results to this study were also reported, wherein sequential fermentation of *M. pulcherrima* and *S. cerevisiae* resulted in higher acetic acid concentrations than fermentation with pure *S. cerevisiae* fermentation [34,35].

### 3.2. Varietal Thiols in Produced Wines

**Figure 1:**
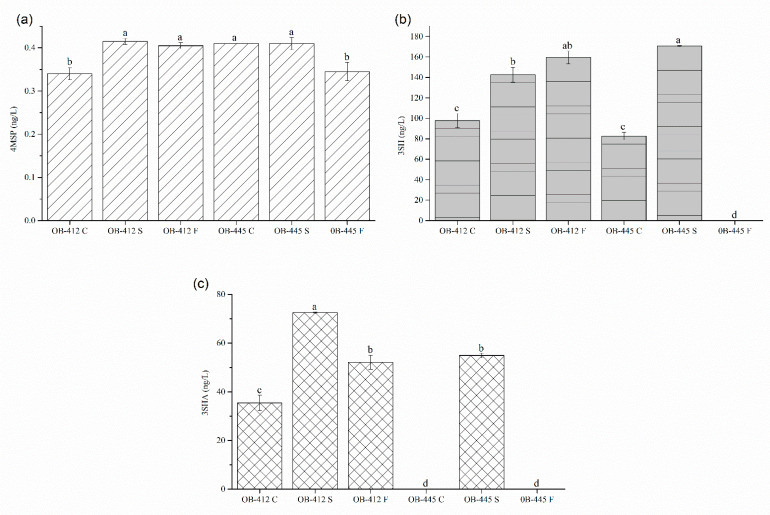
Concentrations of varietal thiols in analyzed Graševina wines: (**a**) 4MSP (**b**) 3SH and (**c**) 3SHA. Different letters indicate significant differences between wines (Tukey’s test, *p* < 0.05). Abbreviations: OB-412 and OB-445 represent the two grape clones used; C- control variant, fermentation with *S. cerevisiae* Lalvin Sensy; S- fermentation with *S. cereviasiae* Sauvy yeast; and F- sequential fermentation of *M. pulcherrima* and *S. cerevisiae*.

The concentrations of the three varietal thiols in analyzed Graševina wines are shown in Figure 1. The 3SH was found in the highest concentrations (82.69–170.63 ng/L), followed by 3SHA (traces- 72.46 ng/L) and, finally, 4MSP (0.34–0.42 ng/L). In general, these concentrations are quite low compared to those found in wines from other grape varieties, especially Sauvignon blanc. For example, according to the review by Roland et al. [36], 3SH concentrations of up to 19,000 ng/L, 3SHA of up to 2500 ng/L, and 4MSP of up to 400 ng/L have been determined in Sauvignon blanc wines. However, there is scarce literature regarding the aroma of Graševina wines and, to the best of our knowledge, only one study researching the presence of varietal thiols in this grape variety. Šuklje and Čuš [37] studied the aroma composition of commercial and microvinificated Welschriesling (Graševina synonym) wines from Slovenia and confirmed the presence of varietal thiols in these wines as well; however, the concentrations they found were higher than those in our study. For example, the concentrations of varietal thiols in commercial Slovenian wines ranged from 362.1–1056.5 ng/L, 198.5–349.7 ng/L, and 3.48–8.80 ng/L for 3SH, 3SHA, and 4MSP, respectively. Additionally, in small-scale produced wines, values of 713.2–983.2 ng/L, 175.8–323.5 ng/L, and 3.67–5.59 ng/L were found for the same compounds. In general, the concentration of varietal thiols in wine is influenced by numerous factors other than the grape variety, such as viticultural practices, climatic conditions, grape ripeness, winemaking practices (yeast strains used, temperature of alcoholic fermentation, oxygenation, etc.), and, finally, storage conditions (described in detail by Roland et al. [36]). Moreover, a wide range of concentrations, from traces to extremely high levels, of individual thiol has also been found in Sauvignon blanc wines, despite the fact that this grape variety is thiol-rich [38,39]. Nevertheless, their sensory thresholds are also very low; namely, 0.8 ng/L, 4.2 ng/L, and 60 ng/L for 4MSP, 3SHA, and 3SH, respectively [4], which means that in most cases these compounds have a significant impact on the varietal character of wine. Moreover, by calculating the “odor activity value” (OAV), the previous statement could be additionally confirmed. Generally, OAV represents the concentration/threshold ratio for each compound, and values higher than 1 imply potentially active odorants [40]. In the case of produced Graševina wines, it could be concluded that 3SH (OAV 1.38–2.66) and 3SHA (OAV 8.44–17.25) contribute grapefruit and passion fruit nuances to their aroma, while 4MSP has no influence, although it was demonstrated that, at lower concentrations, it contributes to the fresh character of the wine [41].

When looking at the differences in concentrations between the wines produced, lower concentrations of 4MSP were determined in the OB-412 C and OB-445 F samples, while the concentrations in the other wines studied were slightly higher than those that were previously mentioned and quite similar to each other. Clearer differences were found for 3SH, wherein higher concentrations were found in the sample of OB-445 S, followed by OB-412 F and OB-412 S. Moreover, this compound was not detected in wine OB-445 F. Finally, the highest concentration of 3SHA was found in the OB-412 S sample, followed by OB-445 S, OB-412 F and OB-412 C, while it was not detected in the OB-445 C and OB-445 F samples. This suggests that Sauvy yeast is the most prominent in the release of thiols, since the highest concentrations of each thiol were found in wines fermented with this yeast (alone or in combination with *M. pulcherrima*). According to the technical data sheets, Sauvy yeast is characterized as a thiol releaser, especially of 4MSP [42]. This could be a possible explanation for the results obtained, wherein only 4MSP was detected in all six wine samples analyzed, while sequential fermentation of *M. pulcherrima* and *S. cerevisiae* (treatment F) had limited effect on the release of 3SH and 3SHA, especially in the case of clone OB-445. The only significant effect of sequential fermentation was observed in the case of clone OB-412 and the 3SH concentration. Ruiz et al. [15] demonstrated that fermentations involving *M. pulcherrima* resulted in lower concentrations of 3SH and 3SHA compared to pure *S. cerevisiae* fermentation. They only observed a difference in the case of 4MSP, where 6.4-fold higher concentrations were detected in wines produced by sequential fermentation of *M. pulcherrima* and *S. cerevisiae*. On the other hand, Sadoudi et al. [33] observed a similar effect with respect to 3SH and 3SHA concentrations during the mixed-simultaneous inoculation of *M. pulcherrima* and *S. cerevisiae* strains; however, in their case, the concentration of 4MSP also decreased. Possible reasons for the previously described differences could be due to the use of different inoculation strategies, as Ruiz et al. [15] performed a sequential fermentation and Sadoudi et al. [33] performed a coinoculation of *M. pulcherrima* and *S. cerevisiae*. Moreover, 3SHA was not detected in wine OB-445 C that was fermented with Lalvin Sensy yeasts (treatment C), which indicated the limited ability of this yeast to convert 3SH to 3SHA.

Finally, regarding the variations between the clones, the most important differences were observed for 3SH and 3SHA, with the clone OB-412 characterized by higher concentrations of these two thiols, especially 3SHA. On the other hand, both clones had similar concentrations of 4MSP, suggesting a limited influence of clone selection on this compound. Previous studies have also found differences between wines produced from different clones of other grape varieties. Most of these studies confirmed differences in the concentrations of 3SH and 3SHA associated with the different grape clones used. For example, Šuklje et al. [7] showed differences in the concentration of the aforementioned thiols in wines produced from two different Sauvignon blanc clones. Chen et al. [43] investigated the influence of yeast strain, commercial enzymes and nutrient additions on the concentrations of enantiomers of 3SH and 3SHA in wines produced from five different clones of the same variety of grapes used in the previously mentioned study. The results showed that grapes from different clones had a significant influence during fermentation beyond the effect of the yeast or additives tested. In addition, a previous study [4] concluded that thiol precursor concentrations differed between the five Sauvignon blanc clones studied. Moreover, these authors found that the concentration of 3SH in the wines also varied between the clones; however, the trends were not the same as those found for the precursors. Finally, our recent study [8] also confirmed this hypothesis and showed that the clone of the Pošip grape significantly affected the concentrations of all three varietal thiols.

Table 2 represents the results (F- and p-values for clone and yeast effect and clone*yeast interaction) of the factorial analysis of variance (ANOVA). Only the clone effect on the concentration of 4MSP was not significant (*p* < 0.05). This means that the concentration of 4MSP was similar for both clones analyzed. On the other hand, both the yeast effect and the clone*yeast interaction were significant, which implies that different yeasts lead to different concentrations of this thiol. Moreover, the concentration of other analyzed thiols (3SH and 3SHA) was significantly affected by clone, yeast and clone*yeast interaction, respectively, implying that all these variations modulate their final concentrations in wine. However, before general conclusions can be drawn, further studies are needed to confirm these observations by involving more clones and yeast strains.

### 3.3. Sensory Analysis

**Figure 2:**
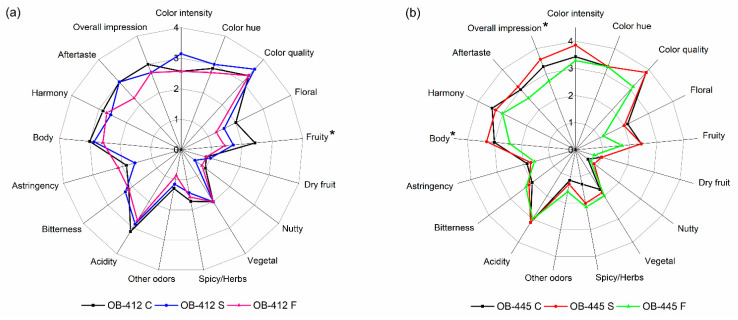
Quantitative descriptive analysis of produced wines: (**a**) clone OB-412; (**b**) clone OB-445. * Statistically significant differences between samples (*p* < 0.05). Abbreviations: C- control variant, fermentation with *S. cerevisiae* Lalvin Sensy; S- fermentation with *S. cereviasiae* Sauvy yeast; and F- sequential fermentation of *M. pulcherrima* and *S. cerevisiae*.

The quantitative sensory analysis showed different trends between produced wines. In the case of clone OB-412 (Figure 2a), wine produced with Lalvin Sensy yeast (control wine) was rated as having higher scores for most of the flavor descriptors (floral, fruity, nutty, spicy/herbs and other odors), as well as for body, harmony and overall impression. In addition, wine fermented with Sauvy yeasts (treatment S) showed higher scores for color parameters (intensity and quality), dry fruit odor and bitterness. Wine fermented with sequential fermentation of *M. pulcherrima* and *S. cerevisiae* (treatment F) was evaluated only as having higher astringency. Nevertheless, statistical analysis (ANOVA) showed that only fruity aroma was significantly different (*p* < 0.05) between tested wines (marked with an asterisk).

In the case of clone OB-445, fermentation with Lalvin Sensy yeasts (treatment C) resulted in a wine scored with higher values for floral odor, astringency, and harmony, while fermentation with Sauvy yeast (treatment S) resulted in higher scores for color intensity, nutty odor, body and aftertaste. Moreover, the latter wine was rated as having the highest overall impression. Finally, the sequential fermentation of *M. pulcherrima* and *S. cerevisiae* (treatment F) resulted in a wine rated as having a higher intensity of vegetal and spicy/herb odors and bitter taste. As in the case of clone OB-412, only a few descriptors (body and overall impression, marked with asterisk) were significantly different between wine samples (*p* < 0.05).

Regarding the published data, Chen et al. [43] also reported lower scores on wine sensory descriptors, such as fruity aroma, body, and bitterness, in wines in which *M. pulcherrima* was involved in sequential fermentation compared to pure *S. cerevisiae* fermentation. Moreover, as in the case of the clone OB-412, Hranilovic et al. [44] showed that fermentation with *M. pulcherrima* resulted in higher astringency than the *S. cerevisiae* control. In contrast to the previously mentioned studies and the trends estimated in this work, some authors showed higher fruity and floral aromas as well as overall impression in the sequential fermentation with *M. pulcherrima* than in the control that used *S. cerevisiae* [18,19]. In addition, Ruiz et al. [15] concluded that higher scores for Verdejo typicity wines were due to the higher content of thiols, especially 4MSP, which is released to a greater extent in wines fermented with *M. pulcherrima*.

Finally, the ranking tests were performed to investigate the panel’s preferences between the wines. Here, the wines of each clone (OB-412 and OB-445) were tested separately, and the panelists were asked to rate the samples in terms of overall impression, with lower scores corresponding to higher preference. The results are presented in Table 3.

As shown in the table, the OB-412 C wine received the lowest score, while the other two wines had similar scores. Of the OB-445 clone wines, the wine fermented with Sauvy yeasts (OB-445 S) obtained the lowest score, followed by OB-445 C and, finally, OB-445 F. In both cases, the wine fermented with sequential fermentation of *M. pulcherrima* and *S. cerevisiae* received the highest score, which means that these wines were the least accepted by the panelists. This is consistent with the results of the quantitative sensory analysis described previously. However, according to the Friedman test, no differences were estimated between the samples, since the calculated *F* values for wines OB-412 and OB-445 are lower/equal (5.43 and 6.00, respectively) than the critical value for three samples and seven judges (6.00).

## 4. Conclusions

The results of this study confirm the presence of varietal thiols in Graševina wines, although the concentrations determined are relatively low compared to thiol-rich grape varieties. However, the results also suggest that their concentration could be modulated by the clone and, in particular, by yeast selection. In this regard, the clone OB-412 is characterized by higher concentrations of varietal thiols, especially 3SH and 3SHA. With respect to the yeasts used, higher concentrations were observed in wines fermented with pure *S. cerevisiae* Sauvy yeasts, while sequential fermentation of *M. pulcherrima* and *S. cerevisiae* affected only the concentrations of 4MSP. Moreover, the sensory analysis showed that fermentation with pure *S. cerevisiae* also resulted in wines that had higher scores for desirable sensory characteristics, such as fruity and floral odor, body and overall impression. The obtained results suggest that clonal selection and yeast strain, as well as their interaction, could have an important role in modulating wine aroma.

## Figures and Tables

**Table 1:** Physicochemical parameters of Graševina wines produced of grape from two clones and subsequent alcoholic fermentation with three yeast strains, during vintage 2020.

Parameter	Clone OB-412			Clone OB-445		
	C	S	F	C	S	F
Alcohol (vol%)	12.8 ± 0.1 b	13.0 ± 0.0 a	12.7 ± 0.1 b	13.1 ± 0.1 a	13.1 ± 0.1 a	12.7 ± 0.0 b
Total extract (g/L)	20.9 ± 0.2 b	22.4 ± 0.1 a	19.0 ± 0.1 a	22.2 ± 0.1 a	21.4 ± 0.1 a	19.6 ± 0.1 b
Reducing sugars (g/L)	<1.0 ± 0.0 c	<1.0 ± 0.0 c	<1.0 ± 0.0 c	1.3 ± 0.1 b	1.6 ± 0.1 a	1.5 ± 0.1 a
pH	2.82 ± 0.01 b	2.81 ± 0.01 b	3.05 ± 0.01 a	2.82 ± 0.02 b	2.81 ± 0.00 b	3.00 ± 0.01 a
Titratable acidity * (g/L)	7.10 ± 0.01 a	6.76 ± 0.05 b	6.33 ± 0.02 b	7.05 ± 0.01 ab	7.24 ± 0.03 a	7.05 ± 0.05 ab
Volatile acidity ** (g/L)	0.41 ± 0.01 a	0.32 ± 0.02 b	0.42 ± 0.01 a	0.32 ± 0.02 b	0.24 ± 0.03 c	0.46 ± 0.01 a

* tartaric acid and ** acetic acid equivalents. Concentrations are expressed as mean ± standard deviation (*n* = 3). Different letters indicate significant differences between wines (Tukey’s test, *p* < 0.05). Abbreviations: C- control variant, fermentation with *S. cerevisiae* Lalvin Sensy; S- fermentation with *S. cereviasiae* Sauvy yeast; and F- sequential fermentation of *M. pulcherrima* and *S. cerevisiae*.

**Table 2:** Summary of *F*- and *p*-values of ANOVA for clone and yeast effects and clone*yeast interactions on varietal thiols in wine.

	Clone (dF ^a^ = 1)		Yeast (dF = 2)		Clone*Yeast (dF = 2)	
	F-Value	*p*-Value	F-Value	*p*-Value	F-Value	*p*-Value
4MSP	0.05	0.83	11.84	<0.01	26.89	<0.01
3SH	282.48	<0.0001	274.26	<0.0001	379.95	<0.0001
3SHA	1170.83	<0.0001	761.29	<0.0001	94.48	<0.0001

^a^ dF- degrees of freedom. Two clones (OB-412 and OB-445) and three yeast strains were employed (*S. cerevisiae* Lalvin Sensy, *S. cereviasiae* Sauvy, and sequential fermentation of *M. pulcherrima* and *S. cerevisiae*).

**Table 3:** Ranking test results.

	Rank Sum	Mean	F (Friedman)
OB-412 C	9	1.29	5.43
OB-412 S	16	2.29	
OB-412 F	17	2.43	
OB-445 C	13	1.86	6.00
OB-445 S	10	1.43	
OB-445 F	19	2.71	

Abbreviations: OB-412 and OB-445 represent two grape clones used; C- control variant, fermentation with *S. cerevisiae* Lalvin Sensy; S- fermentation with *S. cereviasiae* Sauvy yeast; and F- sequential fermentation of *M. pulcherrima* and *S. cerevisiae*.

## Data Availability

Data is contained within the article.
